# Developing and Validating a Competency Framework for Non-clinical Simulation Operations Specialists

**DOI:** 10.7759/cureus.100408

**Published:** 2025-12-30

**Authors:** Anjali Jagannathan, Refka Al-Bayati, Krystina M Clarke, Julia Micallef, Timothy Willett, Nick Wattie, Adam Dubrowski

**Affiliations:** 1 Health Sciences, Ontario Tech University, Oshawa, CAN; 2 Medical Education and Simulation, Simulation Canada, Ottawa, CAN

**Keywords:** competency-based education, competency framework, healthcare simulation, simulation-based education, simulation operations specialist

## Abstract

Background: Simulation-based education (SBE) is essential for developing and maintaining clinical skills, yet its effectiveness is partially contingent on simulation operations specialists (SOS) who provide technical, pedagogical, and safety support. Traditionally, SOS roles have been filled by clinicians, but healthcare workforce shortages have prompted simulation centres to rely on informal, on-the-job pathways to train non-clinicians as SOS. This approach has raised concerns regarding workforce readiness and highlights the absence of structured training pathways. To address this gap, we developed and validated a competency framework explicitly tailored to entry-level, non-clinical SOS to inform the development of structured training pathways.

Methods: A mixed-methods design guided by participatory action research (PAR) was used to guide this work. This study followed Batt et al.’s six-step model to develop and validate the competency framework. Methods included a narrative review, artificial intelligence (AI)-supported competency generation, semi-structured interviews, a card-sorting exercise, survey-based validation, and focus groups.

Results: This study produced a validated competency framework for non-clinical SOS training consisting of 36 competencies across three technical pillars: (i) Simulation Technology (SIMTECH); (ii) Educational Principles (EDUPRI); and (iii) Safety (SAFE), plus a General Competencies (GEN) pillar aligned with transferable knowledge, skills, and attitudes (KSAs).

Conclusion: This study provides the first validated competency framework tailored for entry-level, non-clinical SOS, grounded in both theory and real-world perspectives. The final framework offers a foundation for curriculum developers, employers, and certification bodies, and informs the development of accessible training pathways for non-clinicians entering the simulation operations field.

## Introduction

Simulation-based education (SBE) plays a critical role in developing and maintaining clinical skills [1]. The effectiveness of SBE is partially contingent on simulation operations specialists (SOS), who support the technical, pedagogical, and safety aspects of simulation delivery [2]. Traditionally, SOS positions have been filled predominantly by clinicians who undergo training to transition into simulation facilitation and education roles [2]. These training initiatives are often embedded within established professional development, faculty development, or certification programs. However, in the context of workforce shortages, many simulation centres have relied on informal, on-the-job training to prepare non-clinicians as SOS [3], hereafter referred to as non-clinical SOS. The training needs of non-clinical SOS differ substantially from clinical SOS, as entry-level, non-clinical personnel require foundational preparation in healthcare systems and interprofessional communication, alongside simulation technology, operations, pedagogy, and safety, areas typically outside their prior education and experience. At present, no formal standardized pathways exist to support the training and integration of entry-level, non-clinical SOS trainees into the simulation ecosystem, raising concerns regarding workforce readiness and professional legitimacy. 

In response, Al-Bayati et al. [4,5] proposed training undergraduate students without clinical backgrounds for SOS roles through competency-based education (CBE) pathways, which emphasize the acquisition of defined knowledge, skills, and attitudes (KSAs) deemed essential for professional practice over time spent in educational settings [6]. Central to CBE, curated sets of KSAs, referred to as competency frameworks, inform such pathways by guiding the development of curricular components [6]. Existing SOS competency frameworks, such as the Certified Healthcare Simulation Operations Specialist (CHSOS®) Examination Blueprint and the SimGHOSTS Capability Framework [7,8], effectively delineate the KSAs essential for professional practice. However, while these frameworks are comprehensive, they do not offer the developmental scaffolding, sequenced competency development, or assessment mechanisms needed to prepare novice learners for autonomous professional practice. Hence, they are not fully accessible to non-clinical undergraduate trainees, nor can they effectively support institutions seeking to develop structured training pathways for entry-level SOS. The lack of universal competency frameworks targeting non-clinical, undergraduate SOS trainees represents a critical gap.

To address this gap, this study sought to develop and validate a competency framework explicitly tailored to non-clinical undergraduate SOS trainees, with the goal of informing the development of structured CBE pathways that produce workforce-ready graduates. 

## Materials and methods

Context

The competency framework described in this study was developed and validated as part of a Master of Health Sciences thesis project conducted at Ontario Tech University, Oshawa, Ontario, Canada [9]. This project focused on co-creating a competency framework for non-clinical SOS, intended to inform the design of the Simulation Technologies, Educational Principles, and Safety (STEPS) minor (https://steps-program.com), a CBE pathway being developed at Ontario Tech University to prepare undergraduate students without clinical backgrounds for SOS roles [4,5]. STEPS represents an early effort to establish a formal educational route for non-clinicians entering the healthcare simulation operations field. Rather than replicating or replacing CHSOS®, STEPS aims to complement existing professional certifications by equipping entry-level, non-clinical SOS with foundational competencies that can support and extend the work of CHSOS-trained staff. Although the full program launch is anticipated for 2028, a pilot cohort of 21 trainees has begun preliminary learning activities, which will provide data to refine and strengthen the framework through ongoing evaluation.

This research received ethical clearance from the Ontario Tech University Research Ethics Board under protocol number 17888, affirming adherence to institutional standards and regulatory requirements.

Study design

This study employed a mixed-methods approach integrating both qualitative and quantitative research methods, including qualitative interviews, structured focus groups, and quantitative surveys, to systematically develop and validate a competency framework for non-clinical SOS. The overarching methodological orientation was participatory action research (PAR), which emphasizes collaborative inquiry and continuous engagement with involved parties to ensure contextual relevance and sustainability [10]. PAR principles informed all phases of this study, though the intensity of engagement increased from Step 3 onwards when broader groups of involved parties were recruited to maximize validity by integrating diverse perspectives. To ensure systematic rigor, the process was structured using a six-step model proposed by Batt et al. [6], which provides a structured approach to developing competency frameworks for health professions. While PAR provided an overarching methodological orientation to guide collaborative inquiry and engagement, Batt et al.’s [6] model ensured systematic development and validation of the competency framework.

Additionally, this study adopted a design-based research (DBR) structure [11], which allowed the development process to occur across micro- and meso-cycles. Micro-cycles involved brainstorming, prototyping, and refinement of the competency framework based on expert feedback, conducted by a core research team of three graduate students with expertise in healthcare simulation and competency-based curriculum design (RA, KC, JM) and the principal investigator (AD), while the meso-cycles consisted of structured quarterly reviews by members of the STEPS program advisory board (AB) (n=20), a steering committee of university-based institutional leadership, simulation centre managers, simulation technologists, SOS professionals, curriculum directors, and national/industry partners (e.g., Simulation Canada and Laerdal Medical). Thus, while the core research team initiated early design phases, ultimately, all decisions were reviewed, refined, and approved by the STEPS AB, ensuring alignment with undergraduate institutional expectations and the broader simulation ecosystem. The following sections provide a detailed discussion of the research activities conducted at each step of Batt et al.’s model [6] and explain how the micro- and meso-cycles informed all major decisions throughout the development and validation process [11].

Process

Step 1: Plan - Identifying the Purpose, Scope, Intended Use(s), and Key Involved Parties

The first step focused on clarifying the framework’s purpose, scope, intended use(s), and key involved parties. Three 120-minute brainstorming sessions were conducted with four researchers (RA, KC, JM, AD). The sessions relied on free-flow brainstorming, allowing participants to openly share ideas before focusing discussions around the framework’s objectives, potential use(s), and key involved parties. Any disagreements were resolved through constructive discussion until a consensus was reached. To create a transparent record of this process, all ideas were documented in a shared Google Document (Google LLC, Mountain View, USA). An inductive thematic analysis of session notes was conducted in Dedoose (Sociocultural Research Consultants LLC, Los Angeles, USA), a cloud-based software application for qualitative and mixed-methods data organization and analysis, following Braun and Clarke’s five-step process: (i) familiarization with the data, (ii) generating initial codes, (iii) searching for themes, (iv) reviewing themes, and (v) defining and naming themes [12]. Two researchers (RA, SS) independently coded the data, compared findings, and resolved discrepancies through discussion until consensus was achieved. An identical thematic analysis process was used to analyze the qualitative data collected in all subsequent steps of this study. The generated themes shaped the initial scope and intended use(s) of the competency framework, and were used to produce a preliminary research plan. 

Following DBR principles [11], the STEPS AB then reviewed these decisions during a scheduled quarterly meeting to evaluate whether the proposed scope and intended use appropriately addressed industry demands, training needs of non-clinical SOS, and university-based institutional constraints. Their feedback informed revisions, and ultimately ensured that the framework would address the shortage of SOS, focus on entry-level non-clinicians, and align with certification pathways (i.e., CHSOS® and SimGHOSTS guidelines) [7,8]. After this initial step, the research team did not participate in any subsequent steps (i.e., focus group, interviews, or surveys) as participants.

Step 2: Identify Contexts of Practice - Generating an Initial Pool of Competencies

To develop a comprehensive understanding of the professional landscape for non-clinical SOS, two complementary approaches were employed.

Narrative review of peer-reviewed and grey literature: Given the limited existing research on the role of SOS, a narrative review was used to synthesize available literature and establish contextual understanding. Searches were conducted in PubMed and Google Scholar on November 29, 2023, using the terms: “simulation technician competencies”, “simulation operations specialist frameworks”, “simulation technician roles”, “simulation technician responsibilities”, “healthcare simulation technicians”, “healthcare simulation operation specialists”, and “simulation competency framework”. The titles and abstracts were independently screened by two researchers (RA, TT), followed by full-text reviews to determine inclusion. Studies that focused on the roles and responsibilities of simulation operation specialists or technicians, as well as those presenting competency frameworks applicable to healthcare simulation, were included. Studies outlining competency frameworks for other, unrelated health professions, as well as studies not published in English, were excluded. Further, to ensure only relevant studies were included, the search was limited to articles published within the last 10 years (2013-2023). Both peer-reviewed and grey literature from both PubMed and Google Scholar were included. Any disagreements between the two reviewers were resolved through discussion until a consensus was reached. 

Each study that met the inclusion criteria was evaluated using the Scale for the Assessment of Narrative Review Articles (SANRA) [13], a validated scoring tool designed to evaluate the quality of narrative reviews and their methodological components. The SANRA scoring (range 0-12) assessed justification, clarity, search transparency, referencing adequacy, reasoning quality, and endpoint relevance. The studies were then analyzed to identify gaps and limitations in existing SOS competency frameworks and to identify specific competencies for SOS to be later refined in subsequent phases of research, producing a robust evidence base for this study.

Artificial intelligence (AI)-generated insights obtained through ChatGPT 3.5 (OpenAI Inc., San Francisco, USA): To complement the narrative review, we strategically employed ChatGPT 3.5 to identify competencies that may exist in practice but remain undocumented in published literature, a common challenge in emerging professional roles. This AI-assisted approach allowed us to cast a wider net while maintaining rigor through human verification and triangulation with peer-reviewed sources. A structured set of predefined prompts was entered into ChatGPT 3.5 on November 29, 2023. Since the knowledge base of ChatGPT 3.5 is limited to materials before 2022, its outputs were treated as an exploratory aid rather than a source of authoritative evidence. Two reviewers (RA, TT) independently screened and refined the results by collapsing synonyms, merging granular KSAs into broader competencies, removing duplicates, and resolving disagreements through discussion until consensus was reached. The complete list of competencies derived from ChatGPT 3.5 and those retained after the screening process were reviewed by the STEPS AB during a scheduled quarterly meeting to ensure accuracy. The date, full list of prompts, and all outputs were documented, archived, reviewed by the research team, and reported to ensure transparency and reproducibility. Table 1 lists all prompts used.

**Table 1:** Prompts entered into ChatGPT 3.5 on November 29, 2023

Prompts
What are the competencies of a simulation technician?
What are the competencies of a simulation specialist?
What are the competencies of a clinical simulation technician?
What are the competencies of a healthcare simulation technician?
What are the competencies of a medical simulation technician?
What are the competencies of a patient simulation technician?
What are the competencies of a simulation technologist?
What are the competencies of a standardized simulation patient technician?
What are the competencies of an educational technology specialist?
What are the competencies of a clinical skills technician?
What are the competencies of a medical simulation technologist?

The resulting competencies were then compiled with those derived from the narrative review into a consolidated pool to be refined in subsequent phases of research. This triangulated approach, combining established literature with AI-assisted exploration, was intended to broaden the search while maintaining methodological rigor through human verification.

Step 3: Explore Practice - Engaging With Involved Parties

To assess applicability and refine the competency pool, a structured focus group was conducted at the 2023 SIM Expo conference held in Ottawa, Ontario, Canada. Using a purposive sampling strategy, nine participants were selected: three healthcare sales representatives (industry), two physiotherapists (clinical educators), two nurses (simulation education specialists), one chiropractor (simulation faculty), and one paramedic (simulation instructor). The purposive sampling strategy enabled in-depth insights from a hand-picked, targeted group of experts representing simulation centres across Canada and diverse professional roles within the simulation ecosystem. The session lasted 90 minutes and was structured into three parts: (i) a 20-minute presentation of the preliminary competency list; (ii) a 60-minute open discussion in which participants critically evaluated and refined competencies; and (iii) a 10-minute closing reflection where participants shared final recommendations.

Three researchers (RA, KC, JM) took field notes on a Google Document, documenting direct quotations, participant interactions, and non-verbal cues, and thematic analysis was conducted in Dedoose using Braun and Clarke’s five-step process [12]. The STEPS AB then reviewed the generated themes to ensure alignment with real-world workforce demands, non-clinical SOS training needs, and university-based institutional constraints. This process revealed critical insights to inform the structure of the competency framework. 

Step 4: Translate and Test - Developing the Competency Framework

Three sub-processes were followed to translate and test the consolidated competency pool identified in step two.

Card-sorting exercise: Five researchers (RA, KC, JM, TT, AD) manually transcribed all competencies onto colour-coded cue cards, with each colour corresponding to the source of the competency (i.e., peer-reviewed literature, grey literature, or ChatGPT 3.5). A collaborative card-sorting methodology [14] was employed to group related competencies into categories and merge redundancies. An open card-sorting approach, in which participants create categories based on their perceptions and organize cards accordingly, was selected due to its flexibility [14]. 

Semi-structured expert interviews: Nine experts (recruited at the 24th International Meeting for Simulation in Healthcare held in San Diego, California, USA, and via referrals) were interviewed (34-120 minutes each), aiming to gather insights to determine how the competency pool should be sorted into categories. Three researchers (RA, KC, JM) conducted the interviews using a structured interview guide informed by the 2023 SIM Expo focus group results, with all notes documented in a shared Google Document. The interview guide emphasized alignment with the CHSOS® Examination Blueprint, as well as pedagogical frameworks used in SBE. Qualitative data were analyzed using Braun and Clarke’s five-step process [12]. Table 2 lists all questions comprising the interview guide. 

**Table 2:** Interview guide used for semi-structured interviews CHSOS®, Certified Healthcare Simulation Operations Specialist; STEPS, Simulation Technologies, Educational Principles, and Safety; SSH, Society for Simulation in Healthcare

Prompts
Based on your experience, can you provide an overview of the CHSOS® certification exam?
In your opinion, how important is it for us to structure STEPS to align with CHSOS®? Why?
Are there specific guidelines or recommendations that we should pay attention to when designing the competency framework?
What are the key competencies or skills that the CHSOS® certification exam aims to assess that we should pay particular attention to?
Technology: Are there any specific simulation technologies or methodologies emphasized in the certification exam?
Pedagogy: Are there any specific simulation-specific pedagogical principles emphasized in the certification exam?
Assessments (of students) and evaluations (of programs): How does the SSH certification address the assessment and evaluation of learners in a simulation setting?
Are there resources, such as templates or case studies that can aid in the development and implementation of the framework?
Are there any planned updates or changes to the CHSOS® exam that should be considered?
What is your idea of an ideal program to prepare undergraduates for the job of a simulation technician?

Survey-based validation: Invitations to participate in a Google Forms (Google LLC, Mountain View, USA) survey were distributed through professional societies and academic networks such as SimGHOSTS, the Society for Simulation in Healthcare (SSH), the STEPS AB, and affiliated academic programs. For confidentiality reasons, these organizations forwarded the invitation directly to their members, and therefore, the research team was not informed of the total number of invitations sent. As a result, a precise response rate could not be calculated. A total of 23 participants responded, representing multiple roles (operations staff, educators, managers) from Canada and the United States. Participants rated each competency’s relevance to the non-clinical SOS role using a five-point Likert scale ranging from one (minimal relevance) to five (maximum relevance). In addition, three participants who had previously completed the CHSOS® exam provided qualitative feedback on how each competency aligned with certification requirements, ensuring the framework’s alignment with professional standards. All survey responses were exported to Microsoft Excel (Microsoft Corp., Redmond, USA) for statistical analysis, where key metrics were calculated to guide decisions regarding the retention, modification, or removal of competencies. Competencies achieving a mean score of ≥ 3.5 and SD ≤ 1.0 were retained [15], while others were flagged for further discussion. 

Step 5: Report - Sharing and Validating the Competency Framework

A focus group with conference attendees at SimGHOSTS 2024 (Indianapolis, Indiana, USA) (n=20) was conducted to validate the competencies with a broader group of involved parties. Participation was open to all interested attendees, ensuring diverse input. The session began with a 30-minute presentation delivered by one researcher (RA), which covered the framework’s purpose, scope, intended use(s), development processes, and results from prior steps, followed by a 90-minute interactive session in which participants discussed each competency in-depth to determine whether it should be retained in its current form, dropped, or refined to better align with the framework’s objectives. Detailed field notes were taken by one researcher (RA), capturing direct quotations, interactions, and non-verbal cues, and thematic analysis was conducted using Braun and Clarke’s five-step process [12]. Following this step, the framework was reviewed by two researchers (RA, AD) to confirm accurate categorization and map individual competencies to major competency titles. The final framework was evaluated by the STEPS AB to confirm accuracy and alignment with workforce demands, training needs, and institutional constraints. 

Step 6: Evaluate, Update, and Maintain - Developing an Ongoing Review and Refinement Plan

To ensure the long-term relevance, applicability, effectiveness, and sustainability of the competency framework, an ongoing review and refinement plan was developed by the research team [6]. This plan was designed to enable the ongoing evaluation and updating of the overarching structure of the framework and its individual competencies, ensuring continuous adaptation based on changes in professional practice, technological advancements, and industry needs [6]. This approach ensures the framework remains grounded in real-world practice, yet responsive to emerging insights and evolving industry needs. Following this plan, one year after validation, the framework was updated in accordance with feedback from involved parties, including the STEPS AB, and emerging insights into the non-clinical SOS scope of practice.

## Results

Findings and synthesis

Step 1: Plan - Identifying the Purpose, Scope, Intended Use(s), and Key Involved Parties

Through three brainstorming sessions, the research team determined that the framework would be designed for individuals entering the simulation operations field without prior clinical experience and would be used to guide the development of structured undergraduate programs in simulation operations. To align with this purpose, advanced roles requiring clinical experience and managerial responsibilities would be excluded. Ultimately, the competency framework would provide a foundation for structured CBE pathways and define a clear training route for non-clinicians seeking to enter the simulation operations field, expanding access to individuals from diverse, non-healthcare backgrounds. Further, by defining the KSAs essential for professional practice, the framework would ensure entry-level, non-clinical SOS meet a consistent standard of proficiency, contributing to workforce readiness and professional legitimacy. Additionally, this framework would be designed to align with industry standards such as the CHSOS® and SimGHOSTS guidelines [7,8].

The third session explored the practical application of the competency framework, its role in educational and professional settings, and the key involved parties. The research team determined that the framework would serve two primary functions: (i) educational: guiding curriculum design; and (ii) professional: serving as a reference for institutions, employers, and certification bodies. Involved parties would include trainees, industry experts, and professional organizations such as Simulation Canada, the SSH, and SimGHOSTS. The researchers anticipated that these involved parties would offer valuable insights into essential competencies, training priorities, and workforce expectations, ensuring the framework reflected industry needs.

**Figure 1:**
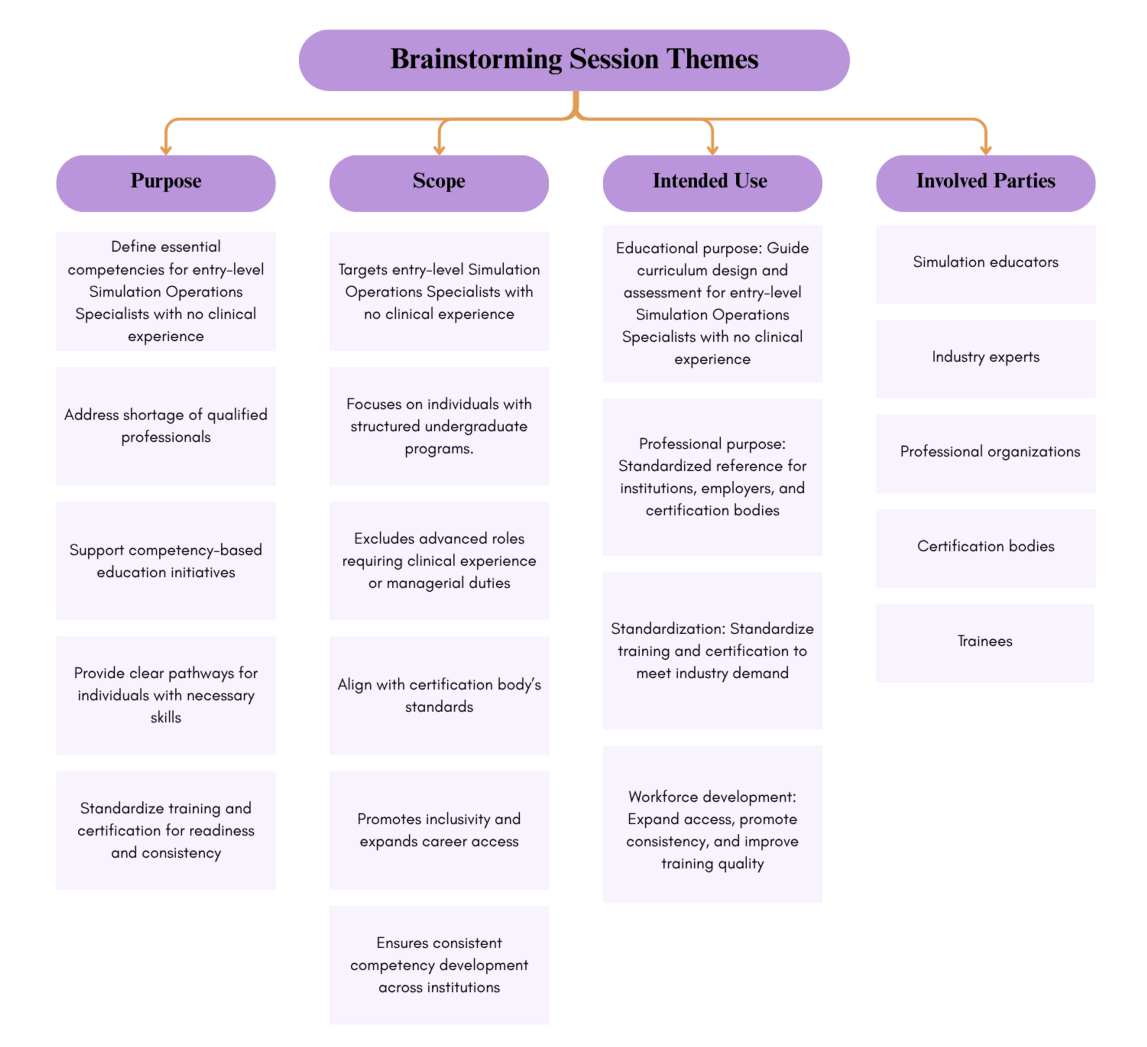
Main themes derived from brainstorming sessions

The themes from the three brainstorming sessions are depicted in Figure 1. All themes were reviewed and confirmed by the STEPS AB during a scheduled quarterly meeting.

Step 2: Identify Contexts of Practice - Generating an Initial Pool of Competencies

Following title and abstract screening, a total of 17 studies met the inclusion criteria for the narrative review. The SANRA scores of the included studies ranged from 8 to 12, confirming adequate methodological rigor [13] (see Appendix A for a table summarizing all studies derived from the narrative review). These studies provided critical insights into existing competency frameworks, highlighting both strengths and gaps in the current healthcare simulation operations training landscape. 

Notably, three key competency frameworks were identified: (i) Roche et al.'s [2], which identified 59 competencies from job postings and 65 through interviews with simulation experts, managers, and educators; (ii) the CHSOS® Examination Blueprint [7], which outlined 35 competencies across five domains; and (iii) the SimGHOSTS Capability Framework [8], which grouped 31 competencies into eight categories. While these three key competency frameworks provide critical foundations for educational and professional development in simulation operations, they do not offer developmental scaffolding, sequenced competency development, or assessment mechanisms, making them largely inaccessible to non-clinical undergraduate trainees and unsuitable for guiding the development of structured undergraduate programs in simulation operations. These findings validated the need for a competency framework targeting entry-level, non-clinical SOS, strengthening the conceptual foundation for this research.

A total of 190 specific, individual competencies were compiled from the three key sources, which were then reviewed and deduplicated, resulting in 123 competencies. Complementing the narrative review, the AI-driven competency identification generated a total of 154 competencies across 11 unique job titles. These were reduced to 19 unique competencies after merging and deduplication. This resulted in an initial consolidated pool of 142 competencies (see Appendix B). This comprehensive process ensured the framework was informed by established research and enriched with emerging, AI-generated insights. 

Step 3: Explore Practice - Engaging With Involved Parties

The focus group at the 2023 SIM Expo conference generated four themes: (i) practical training; (ii) role differentiation; (iii) workforce/career pathways; and (iv) certification alignment. Importantly, a fifth unanticipated theme emerged regarding recruitment and outreach. The key themes and supporting illustrative quotes derived from this focus group are summarized in Appendix C.

**Figure 2:**
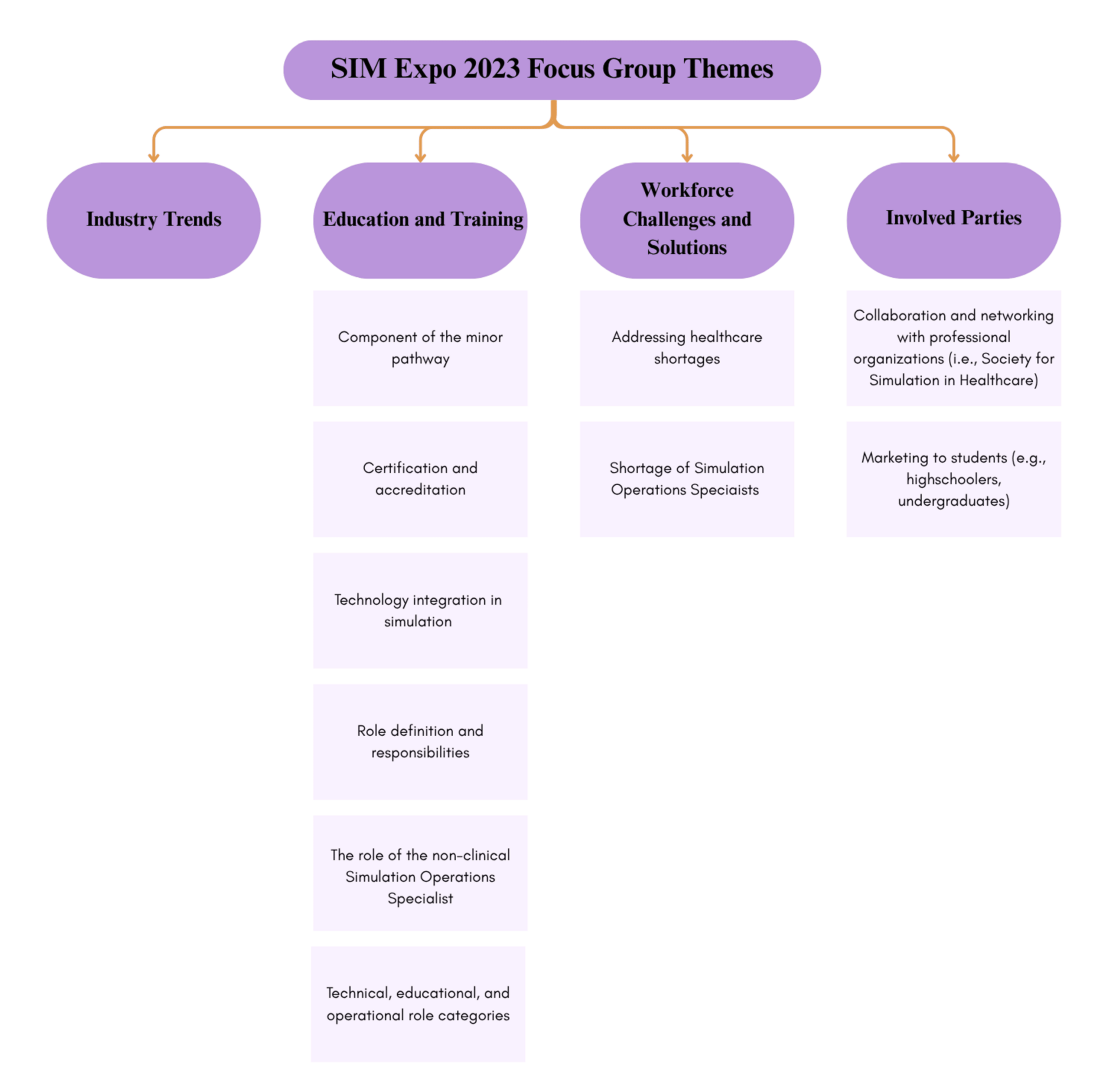
Main themes derived from 2023 SIM Expo conference focus group

Participants emphasized the variability of SOS training, reinforcing the need for standardized hands-on training embedded within curricula. They also discussed the role ambiguity between SOS, educators, and operations staff, and proposed clarifying three role categories: (i) technical (technology operation and troubleshooting); (ii) educational (instructional design alignment); and (iii) operational (logistics and scheduling). Workforce challenges such as burnout, limited advancement opportunities, and role instability were identified, with recommendations for clear career pathways. Certification was seen as essential for legitimacy, with calls for alignment with CHSOS® standards [7]. Finally, outreach strategies, such as targeting high school students, embedding micro-credentials and undergraduate minors, and strengthening recruitment pipelines, were recommended. These results are summarized in Figure 2.

Step 4: Translate and Test - Developing the Competency Framework

The translation and testing phase included three components.

Card-sorting exercise: Through a three-hour, open card-sorting exercise [14], five researchers reduced the 142 competencies to a set of 59, sorted across three categories (Table 3). This represents a 59% reduction, achieved by consolidating redundancies and removing 14 managerial competencies outside the undergraduate, entry-level scope. 

**Table 3:** Preliminary thematic categories and example competencies IT, information technology

Preliminary Category	Representative Competencies
Technology/operational skills	IT troubleshooting, audiovisual support, equipment maintenance, network security
Educational principles	Scenario development, instructional design, simulation-based learning facilitation
Safety/quality improvement/miscellaneous	Universal precautions, safe disposal of potentially hazardous materials, confidentiality

**Figure 3:**
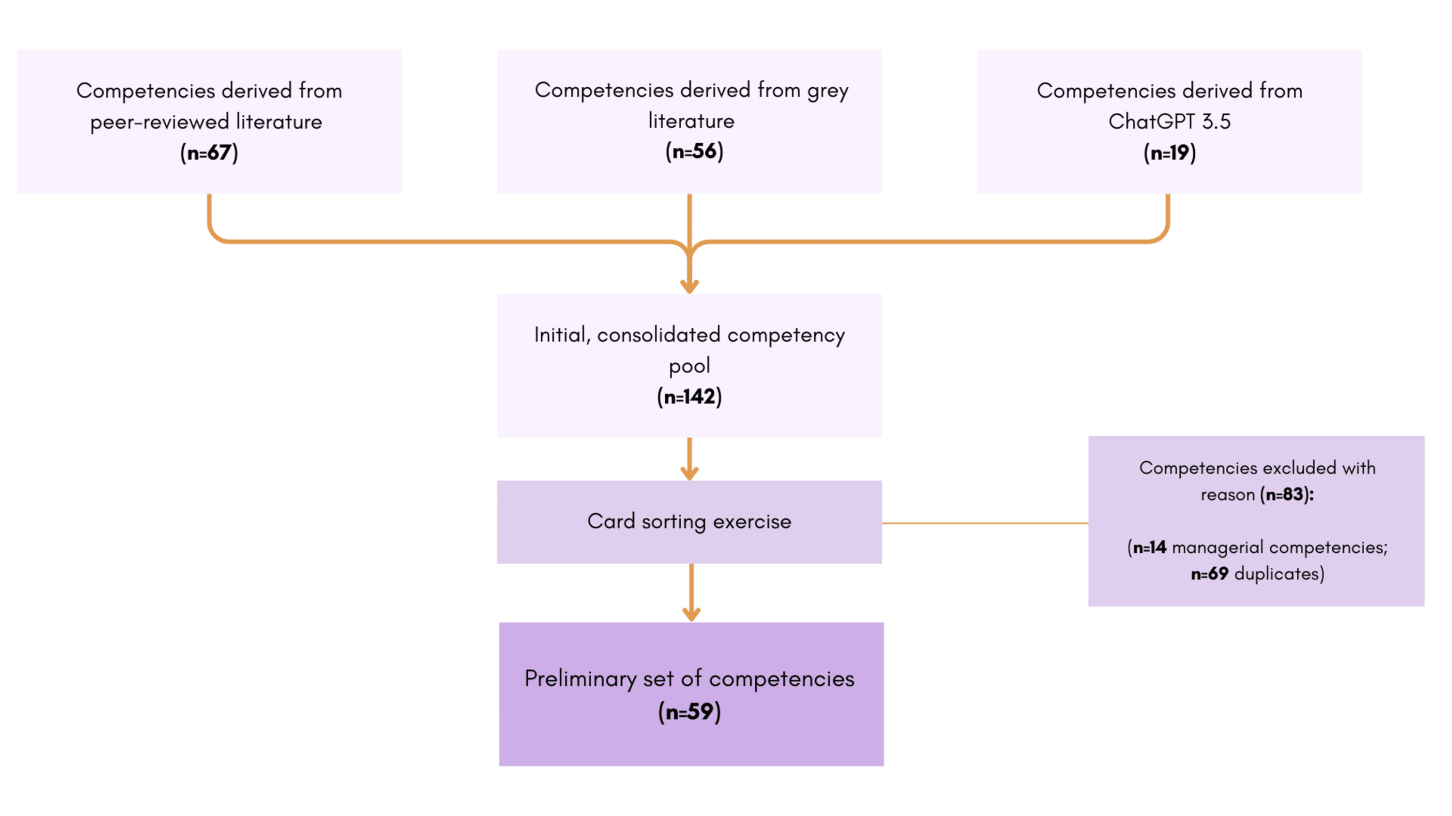
Initial competencies and their reduction achieved through card-sorting exercise

Initially, the research team grouped similar competencies to form preliminary thematic categories and consolidated redundant competencies into broader statements, resulting in the removal of 69 competencies. Additionally, in accordance with the scope and purpose of the competency framework, any competencies related to managerial roles, including project management, budget oversight, and curriculum development, were excluded, which resulted in the removal of 14 competencies. The remaining 59 competencies were retained (presented in Appendix D). Both the initial pool of competencies defined in Step 2 and their reduction leading to the preliminary set of competencies in Step 4 are illustrated in Figure 3. 

**Figure 4:**
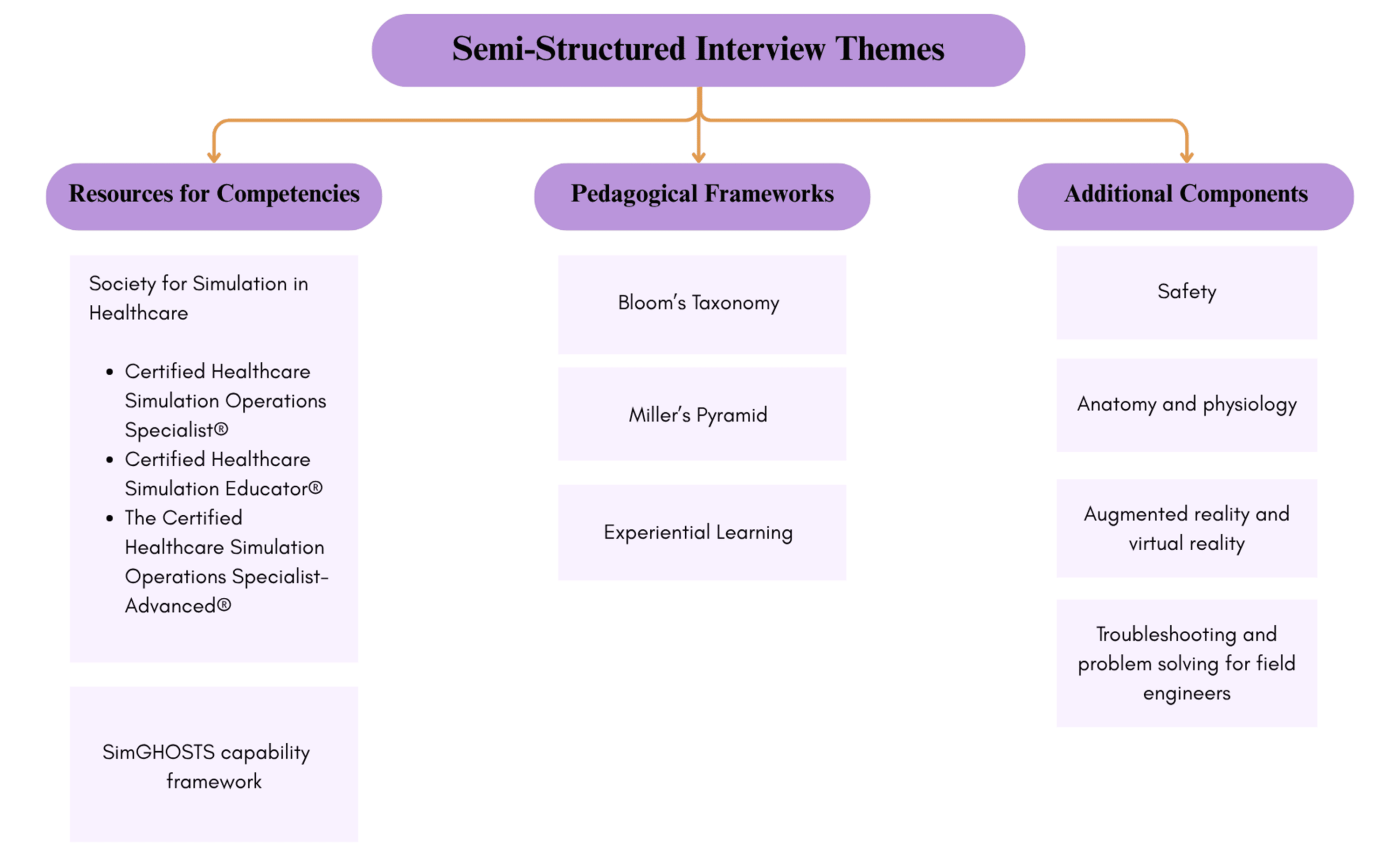
Themes derived from the semi-structured interviews with simulation experts

Semi-structured interviews: Participants in the semi-structured interviews emphasized the importance of aligning the framework with established resources such as the CHSOS® Examination Blueprint [7] and the SimGHOSTS Capability Framework [8], both of which outline technical and operational competencies in healthcare simulation operations. Additionally, participants highlighted the importance of educational frameworks used in SBE due to their capacity to provide structured pedagogical guidance [16,17]. Lastly, safety emerged as a key theme in the interviews, identified by participants as critical for ensuring the authenticity and real-world applicability of SBE. The main themes derived from these interviews are depicted in Figure 4. 

Survey validation: A Google Forms survey was distributed to simulation experts identified through professional societies and academic networks. A total of 23 responded. Confidentiality restrictions prevented the calculation of a response rate. Participants rated each competency on a five-point Likert scale, and three CHSOS®-certified participants provided qualitative feedback on certification alignment. About 77% of competencies met retention thresholds (mean ≥ 3.5, SD ≤ 1.0) [15], while the 13 that did not meet retention thresholds were flagged for further discussion. The complete results from the Google Forms survey are summarized in Appendix E.

Step 5: Report - Sharing and Validating the Competency Framework

Following inductive thematic analysis [12], key themes were derived from the SimGHOSTS focus group. Participants emphasized the importance of prioritizing troubleshooting skills in network and connectivity management and distinguishing between mechanical skills and IT/computer skills. They also suggested removing the videography component from the audiovisual and videography competency, acknowledging that it falls outside the typical scope of the non-clinical SOS role. In addition, participants recommended refinements to wording for clarity and accuracy, such as replacing “cable” in “cable and wireless connectivity” with “wired”. All 59 competencies were either retained in their current form or modified to better align with the framework’s objectives.

To ensure parsimony and minimize redundancies, the resulting competencies were grouped and mapped to major competency titles. To strengthen alignment with the Co-operative Education and Work-Integrated Learning (CEWIL) Canada framework [18], a distinction was introduced between technical competencies (i.e., those tied directly to simulation operations tasks) and general competencies, which CEWIL defines as broad, transferable skills (e.g., communication, teamwork, problem-solving, adaptability) that support learners across real-world professional contexts. Based on this distinction, the framework was organized into three technical pillars: (i) Simulation Technology (SIMTECH); (ii) Educational Principles (EDUPRI); and (iii) Safety (SAFE), and one additional pillar for General Competencies (GEN). This structure preserves the technical precision required of non-clinical SOS while also acknowledging the foundational general competencies that enable adaptability and success in varied professional environments. A comprehensive list of all feedback provided by the SimGHOSTS focus group participants is provided in Appendix F.

Step 6: Evaluate, Update, and Maintain - Developing an Ongoing Review and Refinement Plan

To ensure the long-term relevance, applicability, and sustainability of the competency framework, the research team developed a structured plan for ongoing evaluation and refinement. This plan operationalizes the evidence-informed principles that guided earlier phases of this study, and integrates both formative and summative evaluation processes designed to ensure that the framework remains aligned with evolving workforce demands, technological advances, and educational needs [6]. Furthermore, it emphasizes participatory engagement with involved parties as an essential feature of framework stewardship, ensuring that decision-making regarding revisions is transparent, data-driven, and representative of both academic and professional workforce perspectives.

The plan includes three complementary mechanisms: (i) pilot testing; (ii) annual expert reviews; and (iii) continuous feedback loops. Together, these mechanisms form a cyclical process of evidence collection, analysis, and refinement that supports the framework’s continuous evolution and responsiveness to changing contexts of practice.

Pilot testing: The framework will be implemented across real-world educational contexts, beginning with the STEPS minor at Ontario Tech University (Oshawa, Ontario, Canada) [4,5] to gather empirical evidence of its effectiveness in preparing trainees for professional SOS practice. Subsequent implementation sites will include additional academic and professional training environments to be identified in later phases of research. The pilot testing phase will not only assess student learning outcomes but also evaluate the logistical feasibility of embedding the framework into curricular structures and assessment systems. Effectiveness will be measured using three indicators: (i) competency acquisition rates assessed through practical performance evaluations, faculty observations, and student self-assessments; (ii) faculty usability feedback collected through standardized surveys, interviews, and reflective debriefings; and (iii) alignment with CHSOS® certification outcomes tracked through post-program certification pass rates and exam performance trends. These data points will be triangulated to establish both the pedagogical and operational value of the framework. Trainee progress and competency development will be monitored longitudinally over a two-year period, enabling iterative refinements based on quantitative data and qualitative feedback, with findings informing the next cycle of framework updates.

Annual expert review: Each year, a panel of 12-15 simulation experts representing SimGHOSTS, the SSH, Simulation Canada, the STEPS AB, and academic programs will review the framework to evaluate its continued alignment with workforce needs and technological developments. This panel will identify emerging competency gaps, analyze new trends, and recommend refinements. To maintain methodological rigor, the annual review will employ structured Delphi panels, allowing for consensus-based revisions and transparent documentation of suggested changes. In addition, the review will assess congruence with the most recent CHSOS® Examination Blueprint [7] and other evolving certification requirements. Recommendations generated through this process will be synthesized into an annual update report and shared with academic and industry partners to promote industry-wide consistency. These annual reviews ensure that the framework remains not only relevant but also adaptable to rapid changes in simulation technologies and workforce needs.

Feedback loops: Embedded feedback loops will ensure that input from both academic and industry stakeholders directly informs framework updating and refinement. These loops will operate through three primary channels: (i) annual SOS professional surveys capturing insights from working professionals regarding skill gaps, technological challenges, and emerging areas of expertise; (ii) evaluations by representatives from hospitals, academic institutions, and industry partners, assessing the framework’s applicability and recommending updates; and (iii) quarterly refinement meetings within implementing programs, where faculty, instructors, and program directors review integration outcomes, teaching challenges, and curricular alignment with industry expectations. These feedback mechanisms will ensure continuous bidirectional communication between educators and practitioners, preserving the participatory ethos central to this study’s methodological orientation [6]. Over time, this structure will establish a living system of framework governance capable of adjusting dynamically to new evidence and the needs of involved parties.

Post Competency Framework Validation Updates - Year One

One year following initial validation, the competency framework underwent its first structured revision, aligned with the evaluation plan established in Step 6 of Batt et al.'s model [6], based on data from scheduled quarterly meetings with the STEPS AB, feedback from involved parties, and emerging insights into the non-clinical SOS scope of practice. Redundant and overlapping competencies were consolidated, misclassified competencies were reassigned to ensure accurate categorization within the four domains, and terminology was simplified to enhance clarity and accessibility for both faculty and learners. The review also emphasized removing unnecessary jargon to ensure that the competencies could be readily interpreted by academic and professional audiences alike. These revisions illustrate the practical functioning of the evaluation plan, demonstrating how engagement with involved parties and empirical data directly inform iterative improvement through a transparent, replicable process.

The updated framework now comprises 36 competencies organized within four domains: (i) SIMTECH (11 competencies); (ii) EDUPRI (nine competencies); (iii) SAFE (six competencies); and (iv) GEN (10 competencies). The revision process further strengthened inter-domain coherence by clarifying competency interdependencies and improving cross-referencing between technical and general KSAs. The complete, validated framework is presented in Appendix G and is publicly available at https://steps-program.com/overview. Moving forward, the annual review and pilot evaluation data will serve as the foundation for an ongoing cycle of refinement, ensuring that the framework remains empirically grounded, continuously relevant, and aligned with workforce needs and standards of best practice.

## Discussion

Overview of findings

This study aimed to develop and validate a competency framework for entry-level, non-clinical SOS. These findings advance both the practical preparation of non-clinical undergraduate trainees and the methodological processes used in competency framework development and competency-based curriculum design. Importantly, this competency framework represents the foundational first step in establishing a structured academic pathway for a university-based program that prepares undergraduate trainees to enter the workforce as SOS professionals. By articulating the core competencies required for safe and effective practice, this framework creates the groundwork upon which curriculum, assessment strategies, and professional development structures can be built.

Additionally, this work contributes to a growing body of research on workforce preparation in healthcare simulation operations while clarifying gaps in the existing literature. While Roche et al. [2] identified essential competencies for simulation technicians, their framework assumed clinical backgrounds, limiting applicability to entry-level, undergraduate students without clinical experience. Similarly, while Zafošnik et al. [3] proposed a competency framework for simulation educators, they emphasized instructional responsibilities rather than technical and operational support. Also, since this framework's target population, simulation educators, differs substantially from entry-level, non-clinical SOS, it was not within the scope of our narrative review. The competencies for simulation educators emphasize curriculum development, debriefing, simulation facilitations, and pedagogical design rather than the technical, operational, and safety domains that reflect non-clinical SOS practice [3]. Additionally, this framework was published after the completion of our narrative review conducted in Step 2, and therefore was not available during the initial stages of our framework development. Other existing frameworks, such as the CHSOS® Examination Blueprint [7] and the SimGHOSTS Capability Framework [8], provide valuable benchmarks but do not offer the developmental scaffolding, sequenced competency development, or assessment mechanisms needed to prepare novice learners for the workforce. These existing competency frameworks were also developed through expert consensus rather than transparent, systematic processes. 

Implications

Theoretical and Methodological Implications

This study makes several important theoretical and methodological contributions to competency framework development and validation. First, this study demonstrates the value of utilizing PAR and rigorously applying all six steps of Batt et al.’s model [6], ensuring transparency, replicability, and systematic integration of the perspectives of involved parties. Prior competency frameworks, such as Roche et al. [2], Zafošnik et al. [3], the CHSOS® Examination Blueprint [7], and the SimGHOSTS Capability Framework [8], all revealed important insights regarding the professional scope of practice in healthcare simulation operations, but lacked the structure and consistency of an evidence-based development model. This study offers a replicable approach to utilizing Batt et al.’s model [6] for competency framework development not only in simulation operations but across diverse emerging professions, ensuring that CBE pathways remain aligned with the realities of real-world practice. This approach can be adopted by curriculum developers to create robust competency frameworks in diverse professional fields, ensuring alignment between curricula and workforce demands.

Practical Implications

The complete, validated competency framework also has significant practical implications. First, by explicitly targeting non-clinical undergraduate SOS trainees, this framework provides a foundation to inform the development of structured university-based SOS training pathways for individuals without prior healthcare experience. For curriculum developers, this provides an evidence-based, validated structure to guide the design of undergraduate CBE programs. For instance, the STEPS minor at Ontario Tech University can use the framework as a blueprint for sequencing courses and scaffolding competency development, designing assessment frameworks, and embedding work-integrated learning opportunities [4,5]. Beyond STEPS, the framework can serve as a reference for micro-credentials or certificate programs developed in collaboration with simulation centers and industry partners. By offering an accessible, cost-effective training route for non-clinicians, this pathway can help reduce onboarding costs, alleviate workforce shortages, and allow institutions to flexibly scale staffing based on simulation demands. 

For employers and simulation centres, this framework defines a consistent standard of proficiency for hiring and training entry-level non-clinical SOS. By moving beyond informal on-the-job learning, institutions can use the framework to reduce onboarding times, improve role clarity, and ensure workforce readiness. Simulation centres often face staffing shortages and variable expectations of SOS roles, and adopting a common competency framework can help address these challenges by standardizing expectations.

For certification bodies such as the SSH, this framework offers empirically validated competencies that could inform updates to the CHSOS® Examination Blueprint [7]. By including competencies specific to non-clinical, entry-level professionals, certification pathways could become more inclusive, legitimizing the contributions of non-clinical SOS while maintaining professional standards.

Finally, for trainees, this framework represents an accessible entry point into simulation careers. By defining competencies that can be taught and assessed without prior healthcare experience, and offering developmental scaffolding, sequenced competency development, and assessment mechanisms, it opens doors to a diverse group of non-clinical undergraduate trainees. This democratization of simulation careers may help address workforce shortages while enriching the field with individuals from varied disciplinary backgrounds.

Limitations

While this framework represents a significant advancement, four key limitations of this study must be acknowledged. First, the validation process was geographically concentrated in North America, limiting generalizability to international contexts, which may differ in terms of undergraduate institutional structures, certification standards, and workforce demands. Second, the framework reflects current simulation technologies, and as new modalities such as AI-driven patient avatars, virtual reality, and automation emerge and become more widespread, the framework will require ongoing updates to maintain relevance. Third, the survey sample (n=23) in Step 4 had an unknown denominator, preventing the calculation of a response rate. Additionally, the survey-based validation supports face and content validity, but does not establish construct and predictive validity, which would require further testing after the implementation of structured training pathways. Lastly, the conference-based sampling approach utilized in Steps 3, 4, and 5 may overrepresent highly-engaged simulation experts or users. Importantly, these limitations do not diminish the central contributions of the study; instead, they highlight next steps for ongoing validation and refinement.

Future directions

Future directions emerging from this study involve incorporating perspectives from hiring managers and institutional decision-makers in the ongoing review, updating, and refinement of the competency framework, as these stakeholders ultimately shape how non-clinical SOS roles are defined, integrated, and valued within simulation centres. Second, international validation studies should be conducted to examine the framework’s applicability across diverse healthcare and educational systems, including low- and middle-income countries where SBE is rapidly expanding. Third, longitudinal testing should follow trainees through structured training pathways (e.g., the STEPS minor and other academic or professional programs) and into the workforce, tracking competency acquisition, CHSOS® certification outcomes, and career trajectories to evaluate workforce readiness. Fourth, the framework should be mapped to existing certification pathways to identify areas of overlap and determine where additional competencies may strengthen professional standards. Finally, as simulation technologies continue to evolve, such as the increasing adoption of AI-driven patient avatars, virtual and augmented reality, robotics, and automation, deliberate mechanisms for technological adaptability will be essential. While we addressed this need locally through the iterative update procedures described in Step 6, long-term generalizability and global relevance will require a broader, consortium-based approach to framework maintenance, including periodic reviews, expert panels, and systematic processes for integrating emerging technological competencies.

## Conclusions

This research addressed a critical workforce gap by providing the first validated competency framework tailored for non-clinical undergraduate SOS training, developed and validated following Batt et al.’s six-step model. By democratizing access to simulation operations careers, this framework not only addresses immediate workforce needs, but also enriches the field with diverse perspectives from professionals with non-clinical backgrounds. This framework also serves as a foundation for developing robust competency-based undergraduate curricula, allowing non-clinical SOS to be trained through structured CBE pathways rather than informal, on-the-job learning. Ultimately, this framework addresses growing workforce shortages by producing highly-qualified non-clinical SOS graduates equipped to support healthcare simulation operations, and offers a replicable approach to utilizing Batt et al.’s model for competency framework development across diverse emerging professions.

## Appendices

Appendix A

**Table 4:** Full list of studies derived from the narrative review

Author(s) and Year	Title	Journal/Source	Key Focus	SANRA Score
Roche et al. (2023) [2]	A mixed-methods study identifying the competencies of health care simulation technicians	Simulation in Healthcare	Identifies competencies required by healthcare simulation technicians, such as knowledge, skills, and attitudes, based on job postings and interviews.	12/12
Crawford et al. (2019) [19]	Bug busters: who you gonna call? professional development for healthcare simulation technology specialists	Advances in Simulation	SimGHOSTS' Bug Busters competition trains healthcare simulation technology specialists by showcasing troubleshooting approaches. It encourages collaboration, assesses technical skills under pressure, and promotes "out of the box" thinking, highlighting the complexity of healthcare simulation technology specialist roles.	11/12
Lowther and Armstrong (2023) [20]	Roles and responsibilities of a simulation technician	StatPearls [Internet]	Provides an overview of the roles and responsibilities of simulation technicians, including equipment setup, software programming, and audiovisual support.	11/12
Society for Simulation in Healthcare (2018) [7]	Certified Healthcare Simulation Operations Specialist (CHSOS®) Examination Blueprint	The Certified Healthcare Simulation Operations Specialist	Foundational document for the Certified Healthcare Simulation Operations Specialist exam, outlining the content domains and the specific knowledge, skills, and attitudes expected of an operations specialist at a two-year competency level.	12/12
SimGHOSTS (2019) [8]	Capability Framework for Healthcare Simulation Technology Specialists	The Gathering of Healthcare Simulation Technology Specialists Inc.	Overview of roles, competencies, and responsibilities for healthcare simulation technology specialists. Discusses eight domains of practice and employment levels.	12/12
Coyne et al. (2021) [21]	A review of virtual simulation for assessing healthcare students’ clinical competency	Nurse Education Today	Review of virtual simulations in healthcare education, focusing on clinical competency assessment, debriefing, and challenges.	10/12
Galloway (2009) [22]	Simulation techniques to bridge the gap between novice and competent healthcare professionals	Online Journal of Issues in Nursing	Explores diverse simulation methods, from role-playing to complex simulators, in enhancing healthcare professionals' skills.	11/12
Charnetski and Jarvill (2021) [23]	Healthcare simulation Standards of Best Practice^TM^ operations	Clinical Simulation in Nursing	Establishes best practices and standards for healthcare simulation operations, focusing on planning, personnel, resources, and financial backing.	9/12
Ahmed et al. (2022) [24]	Development of a simulation technical competence curriculum for medical simulation fellows	Advances in Simulation	Focuses on a technical competence curriculum for medical simulation fellows, with hands-on practice and final assessments.	8/12
Motola et al. (2013) [25]	Simulation in healthcare education: a best evidence practical guide	Medical Teacher	Offers a comprehensive guide on simulation education principles, covering feedback, debriefing, curriculum integration, and learning styles.	11/12
Handley and Dodge (2013) [26]	Can simulated practice learning improve clinical competence?	British Journal of Nursing	Examines the use of simulation in nursing education, the integration into curricula, and the challenges in evaluating clinical competence.	9/12
Fanning and Gaba (2007) [27]	The role of debriefing in simulation-based learning	Simulation in Healthcare	Discusses the role of debriefing in simulation, its phases, and facilitator responsibilities in ensuring effective learning outcomes.	9/12
Bailey et al. (2015) [28]	Defining the simulation technician role	Simulation in Healthcare	Investigates the roles and responsibilities of simulation technicians, emphasizing equipment setup, programming, audiovisual support, and maintenance.	12/12
Lyons et al. (2015) [29]	Enhancing the effectiveness of team debriefings in medical simulation	The Joint Commission Journal on Quality and Patient Safety	Offers best practices for team debriefings in medical simulations, focusing on preparation, facilitator roles, and debriefing content.	9/12
Gantt and Young (2016) [30]	Healthcare Simulation: A Guide for Operations Specialists	Hoboken, New Jersey: John Wiley & Sons	Provides essential insights into the evolving role of simulation operations specialists in healthcare. It covers key topics such as curriculum integration, medical terminology, scenario standardization, and professional development.	12/12
Gough and Hamshire (2012) [31]	Supporting healthcare workforce development using simulation and e-portfolios	Proceedings of ePIC 2012, the 10th International ePortfolio and Identity Conference	Focuses on combining simulation scenarios with digital tools like e-portfolios to enhance clinical skill development and student reflection.	8/12
Crawford et al. (2019) [32]	Comprehensive Healthcare Simulation: Operations, Technology, and Innovative Practice	Cham: Springer	This guide outlines the key knowledge, skills, and personnel needed for healthcare simulation operations. It covers core domains for healthcare simulation technology specialists, with best practices, innovations, and industry collaboration strategies.	12/12

Appendix B

**Table 5:** Initial pool of competencies derived from external sources IT, information technology; AV, audiovisual

Initial Pool of Competencies
From Peer-Reviewed Literature
Simulation technology
IT
Audiovisual technology
Communications technology
Social media
General clinical knowledge
Medical equipment
Anatomy and physiology
Medical terminology
Universal precautions
Educational principles
Use of simulation for education
Problem solving
Organizing
Innovation
Adaptable
Planning
Procurement
Inventory management
Competence
Time management
Proactive
Multitask
Resilience
Curriculum development
Strategic development
Prioritize
Project management
Decision making
Teamwork
involved parties management
Diplomacy
Attentive
Leadership
Conflict resolution
IT skills
General technical skills
Maintenance
Moulage
Research
Craft
Videography
Customer centric
Compliance
Confidentiality
General professionalism
Respectful
Gracious
Inclusivity
Assertive
Advocacy
Responsible
Willingness to learn
Open-minded
Self-motivated
Positivity
Crisis management
Presentation skills
Instructional
Role play
Independent
Inquisitive
Diligent
Pedagogic
Report writing
Take initiative
Communication skills
From Grey Literature
Identify the presentation of general medical conditions, injuries, and diseases
Recognize basic anatomical and physiological systems
Identify general healthcare procedures
Identify common medication administration practices
Distinguish among healthcare equipment, supplies, and environments
Differentiate among the roles of healthcare professionals
Differentiate among operating systems and associated compatibilities (e.g., Windows, Mac, Linux, Android)
Apply functional knowledge and terminology for the utilization of network hardware
Apply functional knowledge and terminology for the utilization of AV equipment and software
Utilize web-based (browser-based) applications and information systems
Collaborate with the team to manage technology systems’ security (e.g., physical, network, data)
Differentiate among the capabilities of simulation modalities
Describe the functionalities of equipment used in simulation, including AV equipment, healthcare equipment, and simulation-specific equipment
Apply data asset management strategies
Apply knowledge required to function in different simulation spaces (e.g., equipment limitations, connectivity, air supply)
Demonstrate knowledge of cable connectivity and applications (e.g., ports, inputs/outputs, adapters, dongles)
Demonstrate knowledge of wireless connectivity and applications (e.g., routers, broadcasters)
Configure, setup, and operate simulation technology, including AV equipment, healthcare equipment, and simulation-specific equipment
Apply principles and procedures to identify technical problems/errors and initiate corrective action
Apply principles and procedures to create policy and perform preventive/regular maintenance
Manage reference materials, equipment specifications, maintenance agreements, and warranties
Collaborate to support program sustainability and/or growth (e.g., strategic plan, simulator purchase, technology services)
Facilitate simulation equipment training
Utilize resources effectively and efficiently (e.g., feasible use of money, people, and space)
Communicate and practice safe/recommended use of simulation equipment and environment
Collaborate with the simulation team to manage schedule requests, supply needs, and participant feedback
Utilize safe removal of potentially hazardous materials and supplies
Collaborate with the simulation team to collect and analyze utilization data
Utilize principles of realism as they apply to simulation activities
Recognize how changing aspects of a simulation activity impact reliability and validity
Recognize concepts that impact simulation (e.g., human factors, patient safety, modeling)
Recognize the concepts of managing risks
Implement moulage principles and applications for various materials and settings used in simulation
Provide orientation for involved parties to simulation principles, equipment, and spaces
Support the public relations activities of the simulation program (e.g., tours, community outreach)
Utilize effective communication strategies
Recognize ethical principles and professional responsibilities as they apply to simulation (e.g., integrity, respect, do no harm)
Demonstrate characteristics of leadership in simulation practice
Recognize opportunities for professional development (e.g., conferences, webinars)
Identify trends in simulation and technology practices
Recognize credible resources (e.g., peer-reviewed journals, product manuals)
Assist in research and innovation activities
Recognize principles of instructional design
Collaborate in needs assessment for simulation activities
Collaborate in setting goals and objectives for simulation activities
Collaborate in developing assessment methods and evaluation tools for simulation activities
Collaborate in planning logistics for simulation activities
Collaborate in selecting modalities for simulation activities
Collaborate in determining equipment and supplies for simulation activities
Collaborate in case/scenario design for simulation activities
Collaborate in planning prebrief/brief, debrief, and participant evaluations for simulation activities
Collaborate in pilot testing (dress rehearsal, field test, run-through) for simulation activities
Collaborate in implementing simulation activities for participants
Collaborate in the evaluation and improvement of simulation activities
Recognize principles of interprofessional education
Recognize when to include subject matter experts
From ChatGPT 3.5
Communication
Safety
Adaptability
Technical proficiency
Ethical awareness
Collaboration
Professional development and education
Feedback/debriefing
Professionalism
Problem solving
Teamwork
Maintenance and management
Research and data analysis
Crisis management
Realism
Healthcare knowledge
People skills
Confidentiality
Attention to detail

Appendix C

**Table 6:** Themes and supporting quotes derived from the SIM Expo 2023 conference focus group

Theme	Illustrative Quote
Industry trends	"Technology is evolving rapidly, and many people are not choosing the more advanced options simply because they lack support and guidance in utilizing them effectively. Despite this, we are creating some truly groundbreaking advanced technology." “I wonder if the growing need is actually for a technology simulationist. You have so much with virtual and so forth, you have that in person, the high fidelity mannequins, you've got all these technology pieces, and I think there's going to be an increased need for a full-time technology simulationist.”
Education and Training	
Component of the minor program	“...And I think if there's a technician piece, I like the idea of having a placement. Because I think learning all the theories is great, but I think having that hands-on and any practice running simulations under supervision.” “So if I was to graduate your program and I'm a paramedic and a sim tech. Or, I'm a medical laboratory specialist, so finding those two-year programs at college, coupling with yours. That's the university pathway to take, and that's great.”
Certification and accreditation	“...I'm seeing what few job postings I do see are starting to say they want. Now, this has come in the U.S.,... And now they're saying if you're going to be teaching people simulation, you better be credentialed.” “Yeah, so, but yes, everything's getting credentialed. Everyone that used to be a bachelor is now a master. Everyone with master's is now a doctorate. Everything that didn't have a certification is going to. So this is happening... It's going to be here in five years.”
Role definition and responsibilities	“...where do you see them [simulation assistants] within the sector? Because the pay grade is slightly different. If you put it as an assistant, I don't think anyone will do it because you won't get paid enough.” “You're now talking about like a very basic technician who I can train in a week, like our co-op we're volunteers. They have high school students. Yep, volunteers.“ “But I think, I think it's like, who are you defining as a simulationist? Are you talking about having three roles? a clinician, a tech, and the tech assistant? Or are you thinking about it where they are complementing one other person?”
Role of a simulation technician	“We [simulation technicians] make it look easy and well-planned. You don't see that my tech spends, like, a week. Trialling different models, so that we could make [the simulation] look exactly right.” “Rather than designing it, you're translating it to reality. And saying these are the learning objectives we want to hit. And I'll say, okay, this is the mannequin that's perfect for this, or we can use this room, we should use these technologies, and then how that gets integrated into the design of the simulation. So I think that's a higher level task, as opposed to the simulation assistant.”
Role of a simulation assistant	“You're now talking about like a very basic technician who I can train in a week, like a high, like our co-op we're volunteers.” “Again, the pay grades are slightly different, the clinicians get paid more, but the tech specialists are doing a lot of what I think you're aiming for.”
Technology integration in simulation	“Medtronic or something like that would be exciting to partner with and think through 'cause that is another bridge between healthcare and tech.”
Workforce Challenges and Solutions	
Addressing healthcare shortages	“...You can see how [healthcare workers pivoting to simulation technician positions] that can detract from the crisis we're in with the shortage.”
Shortages of simulation technologists	“We spend a year [training simulation technicians]. And then if they [simulation technicians] leave, because they're also burnt out, you know, that's a problem.”
Networking and Marketing	
Collaboration and networking	“These are people who've done it [build a simulation technology training program]. And we can tap into them and get some, you know, why reinvent the wheel?”
Collaboration with the Society of Simulation in Healthcare (SSH)	"You talked about you taking to the CHSOS blueprint, basically tore that apart to develop the competencies. I think a great end goal is to achieve CHSOS certification and go into the world.”
Marketing to students	“[The program provides] a fallback plan … [If I was] a paramedicine for eight years and then I want something different. I'm not locked into that. I can do something different.”
Marketing the program to high school students	“How would you market high school students to sign up and take this program? That'd be the cool part that I'd be interested in, in seeing your pathway to do that.” “I was thinking from a high school student perspective: this is awesome, you've got a new program, it's in health sciences...”
Marketing the program to undergraduate students	“So if I was to graduate your program and I'm a paramedic and a sim tech. Or, I'm a medical laboratory specialist. So finding those programs at college, coupled with yours [simulation technician training program], and that's the pathway to take, and that's great. I have bills to pay, I don't want to take time out, I don't want to go back to school.” “If we take this piece [simulation program] on as a minor, it at least would let you explore a little bit.” “I have a suggestion for how [the program] could be structured where… So what if you tried to, like thinking about the pay scale, to leverage your students while they're still in their undergrad, offered as a micro-credential that's offered after hours or in their summer break or something like that, and then hire undergrad students while they're still in school.”
Marketing the program as a post-grad diploma	“But if you were to set them up for success, like, again, the number of paramedics that we talk to that say I've been doing this for five years and I don't know, I don't find it rewarding anymore, I feel burnt out, and they have nothing else. No fallback. But this would be great.” “But, what if you were to flip that with the nursing attrition because people are getting in and then getting out… this would be a fallback for them. So you don't lose the talent, you can at least repurpose it.”

Appendix D

**Table 7:** Competencies retained after the card-sorting exercise AV, audiovisual; IT, information technology

Competencies and Thematic Categories
Technology/Operational Skills
Distinguish among healthcare equipment
Anatomy and physiology
Understand common medical terminology
Identify common medication administration practices
Identify the presentation of general medical conditions, injuries, and diseases
Differentiate among the roles of the healthcare professionals
Apply principles and procedures to identify technical problems/errors
Initiate corrective action
Apply principles and procedures to create policy and perform preventive/regular maintenance
Inventory management
Adaptability
Simulation technology/IT skills
Configure, setup, and operate simulation technology: AV equipment
Configure, setup, and operate simulation technology: healthcare equipment
Configure, setup, and operate simulation technology: simulation-specific equipment
Utilize principles of realism as they apply to simulation activities
Feedback/debriefing
Realism
AV technology/videography
Differentiate and assess compatibility across operating systems (Windows, Mac, Linux, Android)
Apply functional knowledge and terminology in utilizing network hardware
Use functional knowledge and terminology for AV equipment and software
Utilize web-based applications and information systems
Collaborate with the team to ensure security in technology systems (physical, network, data)
Differentiate simulation modalities and their capabilities
Describe the functionalities of simulation equipment, including AV, healthcare, and simulation-specific equipment
Implement data asset management strategies
Apply knowledge for functioning in diverse simulation spaces (equipment limitations, connectivity, air supply)
Demonstrate knowledge of cable connectivity and applications (ports, inputs/outputs, adapters, dongles)
Demonstrate knowledge of wireless connectivity and applications (routers, broadcasters)
Educational Principles
Communicate and practice/recommend the use of simulation equipment and environment
Demonstrate the use of equipment
Collaborate in the following instructional design elements for simulation activities: needs assessment
Collaborate in the following instructional design elements for simulation activities: goals and objectives
Collaborate in the following instructional design elements for simulation activities: assessment methods and evaluation tools
Collaborate in the following instructional design elements for simulation activities: logistics
Collaborate in the following instructional design elements for simulation activities: modalities
Collaborate in the following instructional design elements for simulation activities: determine equipment and supplies
Collaborate in the following instructional design elements for simulation activities: case/scenario design
Collaborate in the following instructional design elements for simulation activities: prebrief/brief, debrief, and participant evaluations
Collaborate in the following instructional design elements for simulation activities: pilot test (dress rehearsal, field test, run-through)
Collaborate in the following instructional design elements for simulation activities: implementation to participants
Collaborate in the following instructional design elements for simulation activities: evaluation and improvement
Recognize concepts that impact simulation
Recognize principles of instructional design
Recognize principles of interprofessional education
Safety/Quality Improvement/Miscellaneous
Universal precautions
Utilize safe disposal of potentially hazardous materials
Recognize the concepts of managing risks and hazards
Confidentiality
Recognize ethical principles and professional responsibilities as they apply to simulation (e.g., integrity, respect, do no harm)
Recognize opportunities for professional development
Recognize when to include subject matter experts
Ethical awareness
Willingness to learn
Teamwork: communication skills
Teamwork: time management skills
Teamwork: multitasking

Appendix E

**Table 8:** Survey results with calculated means and standard deviations SD, standard deviation; IT, information technology; AV, audiovisual

Competency	Mean	SD
Simulation Technology: Selection, Use, and Maintenance		
Distinguish among healthcare equipment	4.35	0.88
Anatomy and physiology	3.52	1.12
Understand common medical terminology	4.3	0.7
Identify common medication administration practices	3.65	0.98
Identify the presentation of general medical conditions injuries and disease	3.91	1
Differentiate among the roles of the healthcare professionals	4.04	0.98
Apply principles and procedures to identify technical problems/errors	4.61	0.72
Initiate corrective action	4.43	0.9
Apply principles and procedures to create policy and perform preventive/Regular maintenance	4.13	0.97
Inventory management	4.3	0.88
Adaptability	4.7	0.56
Simulation technology/IT skills	4.52	0.79
Configure, setup, and operate simulation technology: AV equipment	4.48	0.79
Configure, setup, and operate simulation technology: healthcare equipment	4.48	0.67
Configure, setup, and operate simulation technology: simulation-specific equipment	4.65	0.78
Utilize principles of realism as they apply to simulation activities	4.35	0.98
Feedback/debriefing	3.96	1.15
Realism	4.13	1.01
AV technology/videography	4.04	1.11
Differentiate and assess compatibility across operating systems (Windows, Mac, Linux, Android)	3.57	1.24
Apply functional knowledge and terminology in utilizing network hardware	3.87	1.01
Use functional knowledge and terminology for AV equipment and software	3.83	0.98
Utilize web-based applications and information systems	3.96	0.93
Collaborate with the team to ensure security in technology systems (physical, network, data)	3.7	1.29
Differentiate simulation modalities and their capabilities	4.61	0.5
Describe the functionalities of simulation equipment, including AV, healthcare, and simulation-specific equipment	4.43	0.84
Implement data asset management strategies	3.26	1.18
Apply knowledge for functioning in diverse simulation spaces (equipment limitations, connectivity, air supply)	4.61	0.78
Demonstrate knowledge of cable connectivity and applications (ports, inputs/outputs, adapters, dongles)	4.04	1.15
Demonstrate knowledge of wireless connectivity and applications (routers, broadcasters)	3.87	1.25
Educational Principles: Uses in Simulation		
Communicate and practice/recommend the use of simulation equipment and environment	4.57	0.66
Demonstrate the use of equipment	4.61	0.72
Educational principles	4.13	1.1
Collaborate in the following instructional design elements for simulation activities: needs assessment	4.13	1.01
Collaborate in the following instructional design elements for simulation activities: goals and objectives	4.22	0.9
Collaborate in the following instructional design elements for simulation activities: assessment methods and evaluation tools	4	1.09
Collaborate in the following instructional design elements for simulation activities: logistics	4.17	0.94
Collaborate in the following instructional design elements for simulation activities: modalities	4.26	0.86
Collaborate in the following instructional design elements for simulation activities: determine equipment and supplies	4.78	0.6
Collaborate in the following instructional design elements for simulation activities: case/scenario design	3.96	1.07
Collaborate in the following instructional design elements for simulation activities: prebrief/brief, debrief, and participant evaluations	3.83	1.19
Collaborate in the following instructional design elements for simulation activities: pilot test (dress rehearsal, field test, run-through)	4.22	0.85
Collaborate in the following instructional design elements for simulation activities: implementation to participants	3.87	1.14
Collaborate in the following instructional design elements for simulation activities: evaluation and improvement	3.87	1.25
Recognize concepts that impact simulation	4.26	0.81
Recognize principles of instructional design	3.78	1.13
Recognize principles of interprofessional education	3.91	1.08
Simulation for Safety and Quality Improvement		
Universal precautions	4.26	1.01
Utilize safe disposal of potentially hazardous materials	4.57	0.95
Recognize the concepts of managing risks and hazards	4.52	0.9
Confidentiality	4.61	1.03
Recognize ethical principles and professional responsibilities as they apply to simulation (e.g., integrity, respect, do no harm)	4.52	1.08
Recognize opportunities for professional development	4.35	0.83
Recognize when to include subject matter experts	4.61	0.66
Ethical awareness	4.52	1.04
Willingness to learn	4.83	0.39
Teamwork: communication skills	4.91	0.42
Teamwork: time management skills	4.87	0.46
Teamwork: multitasking	4.83	0.49

Appendix F

**Table 9:** Feedback from SimGHOSTS 2024 focus group participants IT, information technology; LMS, learning management system

Competency	Participant Feedback	Conclusion
Network and connectivity management	Participant 1: Not typically within the department's scope; focus should be on connecting machines to the network, not broader network management. Participant 2: Highly relevant due to frequent connectivity issues requiring troubleshooting. Participant 3: Essential competency; involves troubleshooting and effective communication with IT support.	Maintain this competency, emphasizing practical troubleshooting skills and the ability to collaborate effectively with IT professionals.
Web-based applications and security	Participant 1: Already possess this knowledge; not necessary as a standalone competency. Participant 2: The entire simulation center relies on a web-based management system, highlighting its importance. Participant 3: Limited control over security protocols; however, proficiency with web-based LMS is critical.	Divide into two competencies: security, focusing on foundational awareness and protocols at a lower Bloom's taxonomy level, and web-based LMS, emphasizing advanced proficiency and integration at the highest level.
Maintenance and inventory management	Participant 1: All staff should handle inventory, while maintenance could be limited to specific roles. Participant 2: Ideally, one individual should manage ordering and inventory logistics. Participant 3: Graduates should be capable of performing both maintenance and inventory tasks.	Retain as a single competency, incorporating both maintenance and inventory management skills to support flexibility and operational efficiency.
Simulation technology skill	Participant 1: Simulation technology skills are not solely IT-focused; competency should distinguish between mechanical skills and IT/computer-related skills.	Split into two distinct competencies: mechanical skills, including equipment setup and maintenance, and computer/IT skills, covering software management, troubleshooting, and digital tools.
Healthcare and simulation-specific equipment	Participant 1: The current competency is too broad; should be divided into four specific competencies to accurately reflect the range of required skills.	Create four distinct competencies, ensuring a targeted approach to specialized equipment, enhancing precision and skill development.
Audiovisual technology and videography	Participant 1: Avoid combining "audiovisual" with "videography"; focus on capturing sessions for debriefing without editing (for reflective learning purposes). Participant 2: Videography is not a core requirement; beneficial but not essential. Participant 3: Capturing footage is sufficient; no need for editing or altering video content.	Simplify this competency by focusing on audiovisual technology, eliminating "videography," and emphasizing recording for debriefing purposes without post-production requirements.
Simulation equipment operation	Participant 1: Competency overlaps with others; need clarity on whether it applies to in situ (real-world) or simulated environments.	Define this competency more precisely, specifying whether it pertains to operating equipment in situ or simulated settings to reduce redundancy.
Cable and wireless connectivity	Participant 1: Update terminology from "cable" to "wired" to improve clarity and reflect current technological language.	Adopt the term "wired and wireless connectivity" for consistency and precision in describing connectivity skills.
Prebrief, brief, and debrief with feedback	Participant 1: Students should understand how to receive and apply feedback; instructors require more advanced facilitation skills. Participant 2: Not typically within a simulation technologist's role; prebriefing may fit, but debriefing responsibilities should be limited. Participant 3: Good to know, but can be developed on the job if needed by the institution. Participant 4: Clarification needed between "prebrief" and "brief," as they are often used interchangeably.	Position this under Educational Principles. Focus on enabling technologists to participate in (not lead) debriefing sessions. Highlight understanding of technology-related outcomes and identifying technical challenges during simulations.
Needs assessment	Participant 1: Suggests using "collaborate" instead of "design" to align with Certified Healthcare Simulation Operations Specialist (CHSOS®) standards.	Accept the terminology shift to "collaborate", emphasizing teamwork and supporting the move from a solitary technologist role to a more collaborative, team-based approach.

Appendix G

**Table 10:** Complete, validated non-clinical simulation operations specialist competency framework VR, virtual reality The competency framework was created by the authors.

Code	Competency	Competency Description
SIMTECH: Simulation Technology		
SIMTECH 1.0	Technology and equipment compatibility	Demonstrate the ability to select, configure, and integrate compatible simulation technologies.
SIMTECH 2.0	Network and connectivity management	Apply knowledge of wired and wireless networks to ensure secure and reliable system connectivity.
SIMTECH 3.0	Web-based applications and security	Navigate browser-based applications, maintain data security, and apply digital best practices.
SIMTECH 4.0	Simulation modalities and equipment use	Operate and differentiate among various simulation modalities, including VR and task trainers.
SIMTECH 5.0	Identification and use of healthcare and simulation equipment	Identify and appropriately use equipment commonly found in simulation centers.
SIMTECH 6.0	Technical problem solving	Apply structured approaches to troubleshooting technical issues under time constraints.
SIMTECH 7.0	Maintenance and inventory management	Perform preventative maintenance, manage inventory, and coordinate repairs.
SIMTECH 8.0	Simulation system configuration and operation	Configure, run, and monitor integrated simulation systems for realistic scenarios.
SIMTECH 9.0	Audiovisual technology and videography	Use audiovisual tools for scenario capture, playback, and remote streaming.
SIMTECH 10.0	Simulation software navigation	Operate software used in scenario authoring, scheduling, and playback.
SIMTECH 11.0	Technical contributions to prebrief, brief, and debrief	Support simulation facilitation by managing technical tools across all phases of simulation.
EDUPRI: Educational Principles		
EDUPRI 1.0	Communication and advocacy	Demonstrate effective communication skills with learners, faculty, and technical staff.
EDUPRI 2.0	Conducting needs assessments	Support faculty in identifying learning gaps and selecting appropriate simulation modalities.
EDUPRI 3.0	Goals and learning objectives	Align simulation activities with learning goals and define measurable objectives collaboratively.
EDUPRI 4.0	Pilot testing	Participate in scenario dress rehearsals and test runs to identify and resolve technical limitations.
EDUPRI 5.0	Scenario implementation	Facilitate scenario execution through reliable technological support.
EDUPRI 6.0	Evaluation and continuous improvement	Support post-scenario review processes through data collection and feedback tools.
EDUPRI 7.0	Simulation design concepts: realism, fidelity, and validity	Understand and apply concepts of fidelity, realism, and educational validity during simulation design.
EDUPRI 8.0	Instructional design principles	Collaborate with educators to support the use of simulation within structured instructional frameworks.
EDUPRI 9.0	Interprofessional education principles	Support interprofessional simulation activities by enabling smooth technical integration across disciplines.
SAFE: Safety		
SAFE 1.0	System-based safety protocols	Follow and support adherence to institutional safety procedures in simulation settings.
SAFE 2.0	Hazardous materials management	Identify, manage, and safely dispose of potentially hazardous simulation materials.
SAFE 3.0	Risk management	Recognize, mitigate, and report potential risks related to simulation environments.
SAFE 4.0	Human factors in simulation	Apply human factors and ergonomics principles to create simulation environments that are safe, usable, and support effective performance.
SAFE 5.0	Usability testing and evaluation	Conduct technical testing to assess equipment and software usability during scenario development.
SAFE 6.0	Supporting psychological safety in simulation	Recognize the importance of psychological safety and contribute to its maintenance by fostering trust, confidentiality, and emotional awareness in simulation environments.
GEN: General Competencies		
GEN 1.0	Medical terminology for simulation technicians	Understand and apply basic medical terminology relevant to simulation scenarios and interdisciplinary communication.
GEN 2.0	Understanding healthcare professional roles	Recognize the functions and scopes of various healthcare roles to support simulation environments.
GEN 3.0	Professionalism and ethical behavior	Demonstrate accountability, punctuality, and ethical conduct in all aspects of simulation practice.
GEN 4.0	Attention to detail	Maintain precision and accuracy in equipment setup, documentation, and simulation execution.
GEN 5.0	Adaptability and Flexibility	Adjust to evolving simulation requirements, timelines, and unexpected challenges with resilience.
GEN 6.0	Time and Task Management	Prioritize responsibilities and manage time effectively in preparation and during simulation activities.
GEN 7.0	Collaboration and Teamwork	Engage constructively with faculty, learners, and other technicians to support simulation delivery.
GEN 8.0	Communication Skills	Convey and receive information clearly in written, verbal, and digital forms across professional contexts.
GEN 9.0	Initiative and Self-Directed Learning	Demonstrate curiosity, seek feedback, and pursue independent learning opportunities.
GEN 10.0	Respect for Diversity and Inclusion	Promote inclusive simulation environments that reflect diverse perspectives and learner needs.
